# Readiness to manage domestic violence among medical interns – an observational study in a medical college and hospital in India

**DOI:** 10.5249/jivr.v16i1.1867

**Published:** 2024-01

**Authors:** Rumelika Kumar, Lina Bandyopadhyay, Monalisha Sahu, Rabindranath Roy, Bobby Paul, Dipankar Jana, Shuvajit Roy

**Affiliations:** ^*a*^ Department of PSM, All India Institute of Hygiene & Public Health, Kolkata, India.; ^*b*^ Department of Community, Medicine, Medical College, Kolkata, India.

**Keywords:** Domestic Violence, Medical Interns, MIREDS, Health care professionals, Health care preparedness, Medical Education, Public Health

## Abstract

**Background:**

Domestic violence is a deeply entrenched issue in Indian society, with global implications, especially for women's physical and mental health. Healthcare providers play important role in early identification and support of the victims. Medical interns, the future generation of Health care professionals, often acting as primary caregivers are uniquely positioned and expected to recognize and assist victims. This study aims to evaluate their knowledge, attitudes, practices, and readiness to manage domestic violence and its associating factors.

**Methods:**

This cross-sectional study was conducted among 157 medical interns at a Medical college and hospital in West Bengal, India, from December 2022 to February 2023. Simple random sampling was done. Data were collected using a semi-structured questionnaire, Medical Intern Readiness to manage domestic violence scale (MIREDS), validated after adoption from Physician Readiness to manage Intimate partner violence scale (PREMIS). Ethical approval was obtained, and participants gave informed written consent for inclusion. Satisfactory threshold was determined to be more than 50 percent. Data analysis was performed using MS Excel and SPSS software, including descriptive and inferential statistics, with a significance level of p less than 0.05, along with logistic regression analysis.

**Results:**

Only 45.2% of medical interns demonstrated satisfactory knowledge, 54.8% had a satisfactory attitude. Most interns (91.7%) exhibited poor practice in dealing with domestic violence cases, only 31.2% considered themselves ready to manage domestic violence cases. Interns who attend more patients was found to have better attitude. Positive associations were found between knowledge, attitude, and readiness to manage domestic violence cases among doctors.

**Conclusions:**

A substantial proportion of medical interns demonstrated inadequate knowledge, negative attitudes, and poor practice and inadequate readiness to manage domestic violence. Comprehensive training and education with cultural sensitivity training along with more practical exposures are in need to address this issue properly.

## Introduction

Domestic violence is a pervasive issue on a global scale, affecting one in three women worldwide. It remains a longstanding concern, profoundly impacting women's health and overall well-being.^1-3^ It's closely associated with harmful behaviors like substance abuse, risky sexual activity, physical complaints, mental health issues, and even the risk of harm or death.^4-6^

While the Indian Penal Code recognized domestic violence as a criminal offense in 1983,^7^ the enactment of the Protection of Women from Domestic Violence Act in 2005 extended civil protections to victims. This comprehensive legislation defines domestic violence to include various forms of abuse. Yet the incidence of domestic violence in India remains high^8^ Alarming statistics from the National Crime Records Bureau reflect 15.3% increase in reported cases against women in 2021 from 2020. 31.8% of cases came under 'Cruelty by Husband or His Relatives'. Additionally, the National Family Health Survey shows that 32% of ever-married women aged 18-49 in India have faced spousal violence, with only 14 % seeking help, emphasizing the urgent need for increased awareness, intervention, and healthcare involvement.^9^

Healthcare providers play a crucial role in addressing domestic violence. Studies indicate their pivotal role in early identification and response to survivors, particularly in India, where abused women often seek medical care after incidents of violence.^10^ Studies have also shown that women are highly accepting of doctors and other healthcare providers inquiring about abuse and providing support services. Victims often view healthcare professionals as potential sources of support. However, given the complexity of the situation, a nonjudgmental and nondirective approach is imperative. Despite this, doctors' hesitancy to inquire or respond effectively when domestic violence is disclosed contributes to the low prevalence of routine screening. Warning signs such as unexplained injuries, bruising, anxiety, and depression often go unnoticed during clinic visits. Many factors, including inadequate knowledge, training, system support, lack of time, fear of offending patients, and personal discomfort, hinder healthcare professionals from addressing domestic violence effectively.^11-13^ Today the healthcare system in India faces challenges in equipping professionals to respond effectively.

Medical Interns constitute a significant portion of frontline healthcare workers who encounter women affected by domestic violence. They are uniquely positioned to recognize victims, provide sensitive counseling, and offer support. Most victims of domestic violence often seek assistance in emergency departments or outpatient departments (OPDs) for medical management. In these settings, medical interns frequently serve as the primary caregivers. Medical Interns are in the process of gaining hands-on clinical experience and represent the future generation of doctors who will play pivotal roles in the healthcare system. As these intern doctors become specialists and future healthcare practitioners, their understanding and approach to this issue will significantly impact the care provided to victims of domestic violence across diverse medical specialties. Therefore, it is imperative that they possess the appropriate knowledge and readiness to effectively manage cases of domestic violence. By ensuring that intern doctors are well-equipped to recognize, counsel, and support victims, we can significantly improve the quality of care and support provided to these individuals during their time of need. This study aims to evaluate their preparedness in order to improve the overall response to domestic violence within the healthcare system. It is crucial that they possess adequate knowledge and training on the matter. However, research on healthcare workers' knowledge and perceptions of intimate partner violence in India is limited. This study aims to assess the knowledge, attitudes, practices, and readiness to manage domestic violence among medical interns and their associating factors in a teaching hospital in Kolkata, West Bengal.

## Methods 

This is an institution-based cross-sectional study. The study was conducted among 157 medical interns in a Tertiary care medical college and hospital in Kolkata, West Bengal, India. Data was collected from December 2022 to February 2023. Sample size was calculated using Slovin’s formula,^14^ taking error(e) as 5% and total N being 257(total number of Medical interns in the study setting in the session 2022-2023. Simple random sampling with replacement was done to select study participants by random number table method. Interns who gave informed written consent were considered eligible for the study. 

The tool used in this study was adapted from PREMIS (Physician readiness to manage intimate partner violence Survey) questionnaire. A new tool was developed which was named MIREDS (Medical Interns readiness to manage domestic violence survey).

The Physician Readiness to Manage Intimate Partner Violence Survey^15^ was created as a comprehensive measure of physician preparedness to manage IPV among their patients. This instrument has been used to measure IPV knowledge, attitudes, and behaviors among primary care physicians and nurses^16-23^ medical students, nursing students,^24^ dental students,^25^ and pharmacists^26^ in multiple previous studies. 

The adapted questionnaire MIREDS had 45 questions divided into 4 domains- Knowledge (5 questions), Attitude (20 questions), Practice (5 questions), System support (5 questions) and Readiness to manage (10 questions). Socio demographic characteristics as well as training status were also taken. Among 67 items in PREMIS, 45 items had been selected according to objectives of our study. Questions on child abuse, elder abuse were not included. Culturally relevant items related to domestic violence were selected and were modified into a new questionnaire. One experts from each of five specialty was chosen- Social Science, Gender Studies, Public Health, Legal scholar and Community Organization Stakeholder. At first, the adapted questionnaire was evaluated by experts based on four parameters: relevance, clarity, simplicity, and adequacy. Based on their opinion appropriate modifications were done till all five experts reached in unanimous agreement about individual items of the questionnaire. Each of the four domains were then evaluated using the content validity index. Here every expert was asked to mark every items of the questionnaire essential or not essential. Based on this Content validity ratio was calculated for each item. After that Content validity index was calculated for each domains and of the questionnaire. For each domain CVI came as follows – (Knowledge-0.8, Attitude- 0.6, Practice- 0.8, System support- 0.7, Readiness to manage 0.7). For the questionnaire CVI was 0.7.

 A pilot study was done from July 2022 to December 2022 with the questionnaire in a different study setting with same background characteristics among 10% of the sample size. Cronbach's alpha coefficient for each domains were calculated (Knowledge- 0.7, Attitude- 0.8, Practice- 0.6, System Support- 0.7, Readiness to manage – 0.8). Intraclass correlation coefficient of 0.8 and Spearman coefficient of 0.7 showed a high reliability. Overall the questionnaire obtained good internal validity, high reliability and predictive self-reported capacity of medical interns. 

Data were collected using a web-based anonymous questionnaire (Google forms). The Modified BG Prasad scale (May 2022)^27,28^ was employed to assess the socio-economic status (SES) of study participants. For knowledge items, each correct options marked were given a score of 1. Attitude Items were based on 5 point Likert scale which was scored on a scale of 0-4. Practice, system support and preparedness questions were scored as yes-1, No-0. Response to each question was recorded and scored. The total score in each domain was converted into a scale of 1 to 100. Satisfactory threshold was determined to be more than 50 percent.

Data were analyzed using MS Excel 2016 and SPSS version 16.0 software. Descriptive and inferential statistics were performed. The statistical significance level was considered as a p-value<0.05. After exclusion of multicolinearity (variance inflation factor of >5), leverage, outliers and influencers. Univariate logistic regression analysis was performed individually for all four domains (knowledge, attitude, practice and readiness to manage). Multivariable logistic regression analysis was performed with all biologically plausible significant variables found in univariate logistic regression analysis. 

## Results

Among 157 participants mean age was 25.0 ± 1.6 years with median of 25 yrs. (Range 22-29) yrs. Table 1 reveals background characteristics of the study participants. Only 3 interns had attended webinar based training by their own initiative. For the knowledge domain, a significant majority, 78.3%, identified societal issues as the primary cause of domestic violence succeeded by drug/alcohol addiction (67.5%). As for reasons behind domestic violence 82.2% recognized that perpetrators often employ violence as a means of controlling their victims. 91.1% of participants selected frequent injuries as most common warning signs of domestic violence. For the question reasons for not leaving an abusive relationship, the most prevalent response was family pressure, cited by 93.0% of participants, followed by societal pressure, mentioned by 77.7%. Regarding attitude of Medical interns about domestic violence, majority of participants (89.2%) agreed that inquiring about domestic violence was a helpful practice. Additionally, a substantial portion (78.9%) of the participants acknowledged the importance of their response as healthcare professionals when dealing with domestic violence cases. However, 27.4% expressed discomfort when it came to asking about domestic violence, and 29.3% found the act of inquiring about abuse to be humiliating. 

**Table 1:** Distribution of study participants according to background characteristics of study participants (n=157)

Age (in completed years)		Number(%)
	21-23	23(14.6)
	24-26	107(68.2)
	≥27	27(17.2)
**Sex**		
	Male	87(55.4)
	Female	70(44.6)
**Religion**		
	Hindu	126(80.2)
	Muslim	15(9.6)
	Others	16(10.2)
**Socio-economic Status**		
	Upper Class	111(70.70)
	Upper-Middle Class	20(12.73)
	Middle Class	26(16.57)
**Marital Status**		
	Married	7(4.5)
	Unmarried	150(95.5)
**Religious view**		
	Religious	143(91.1)
	Non-Religious	14(8.9)
**Place of upbringing **		
	Rural	79(50.3)
	Urban	78(49.7)
**School Education Medium**		
	Bengali	94(59.9)
	English	45(28.6)
	Hindi	18(11.5)
**Average Number of Patients per Week**		
	<20	12(7.7)
	20-39	25(15.9)
	40-59	41(26.1)
	>=60	79(50.3)
	Total	157

Table 2 illustrates Practices and readiness to manage domestic violence victims among study participants. About practice regarding domestic violence victims, 49.0% of participants had identified new cases of domestic violence in the last 1 month, with the majority identifying between 1 to 5 cases during that time frame. Regarding the steps taken after identifying domestic violence, a significant portion (75.3%) of participants offered psychological support to the affected patients. However, only 32.5% documented the history of domestic violence and physical examination findings.

**Table 2:** Distribution of study participants according to Practices undertaken and readiness to manage domestic violence victims.

Screening Practices(n=157)					Frequency(%)	
I do not currently screen					115(73.3)	
I screen all new patients					1(0.6)	
I screen all new female patients					2(1.3)	
I screen all patients with abuse indicators on history or exam					17(10.8)	
I screen certain patient categories					22(14.0)	
Steps taken after identifying DV victims(N=77*)					Yes	No
Provided basic information about domestic violence					12(15.6)	65(84.4)
Offered psychological support					58(75.3)	19(24.7)
Documented domestic violence history and physical examination findings					25(32.5)	52(67.5)
Assessed the immediate level of danger for the woman					13(16.9)	64(83.1)
Refer the woman to support services available within the community (psychological, legal, shelter, etc.)					13(16.9)	64(83.1)
*(Only 77 of the study participants identified DV victims in last 6 months)							
Readiness to manage	Not ready	Little Ready	Moderately Ready	Fairly Ready	Expert	Total
Identify domestic violence victims	8 (5.1)	94 (59.9)	43 (27.4)	10 (6.3)	2(1.3)	157
Signs or symptoms of DV	6 (3.8)	71(45.2)	50(31.9)	30(19.1)	0	157
Ask appropriate questions about DV	13 (8.3)	86 (54.8)	46 (29.3)	10 (6.3)	2 (1.3)	157
Appropriately respond to DV victims	10 (6.4)	78 (49.7)	49 (31.2)	19 (12.1)	1 (0.6)	157
Help an DV victim assess his/her danger	83 (52.9)	56 (35.6)	13 (8.3)	3 (1.9)	2 (1.3)	157
Document Domestic violence history and physical examination findings in patient’s chart	73 (46.5)	66 (42.0)	11 (7.0)	6 (3.9)	1 (0.6)	157
Developing a safety plan with an DV victims	127 (80.9)	25 (15.9)	2 (1.3)	1 (0.6)	2 (1.3)	157
legal reporting requirements and making appropriate referrals	118 (75.2)	27 (17.2)	7 (4.54)	3 (1.9)	2 (1.3)	157

Scores obtained in each domain by the study participants are shown in Table 3. Notably, only 45.2% of medical interns demonstrated sufficient knowledge about domestic violence. 45.2% medical interns displayed a negative attitude towards domestic violence. Overall, 91.7% exhibited poor practice in dealing with domestic violence cases. Out of the 157 participants, only 31.2% considered themselves ready to manage domestic violence victims. 

**Table 3:** Descriptive of scores obtained in various domains by the participants (n=157).

Domains	Median(IQR)	Range(Min,Max)
Knowledge	40.(37,52)	74(15,89)
Attitudes	67(60,76)	(54.45,99)
Practice	0(0,25)	100(0,100)
Readiness to manage	23(12,31)	78(0,78)
Participants distribution based on scores obtained in various domains		
Domains	Unsatisfactory Number(%)	Satisfactory Number(%)
Knowledge	86(54.8)`	71(45.2)
Attitude	71(45.2)	86(54.8)
Practice	144(91.7)	13(8.3)
Readiness to manage	108(68.8)	49(31.2)

Univariate logistic regression analysis in the Table 4 indicated that interns raised in an urban locality have 1.9 times higher odds of having satisfactory knowledge ([OR] = 1.0, 95% confidence interval [CI] = 1.0-3.6, p-value = 0.04). Interns who reported seeing more patients on a weekly basis had 1.9 times higher odds of having better attitude towards domestic violence (OR = 1.9, 95% CI = 1.0-3.6, p-value = 0.04).Interns with satisfactory knowledge had 2.1 times higher odds of having satisfactory attitude (odds ratio [OR] = 2.1, 95% confidence interval [CI] = 1.1-4.0, p-value = 0.02) and 1.2 times higher odds of having satisfactory readiness to manage domestic violence (odds ratio [OR] = 1.2, 95% confidence interval [CI] = 1.1-2.2, p-value = 0.02). Interns with Satisfactory attitude also had 1.9 times higher odds of having satisfactory readiness to manage domestic violence (odds ratio [OR] = 1.9, 95% confidence interval [CI] = 1.2-3.8, p-value = 0.03). Multivariable regression analysis is given in table5. Three predictor variables for satisfactory attitude regarding domestic violence emerged Religion [OR (95% CI) = 1.8(1.0,3.4); p-value-0.02]; Patients weekly[OR(95%CI) = 2.2(1.3,5.2); p-value-0.04]; Knowledge[OR(95%CI) = 2.0(1.0,3.9); p-value -<0.05]. Two predictor variables for satisfactory readiness to manage domestic violence were knowledge [OR(95%CI) = 1.5(1.3,2.4); p-value -0.04] and attitude [OR(95%CI) = 2.2(1.0,4.4); p-value – 0.02]. Hosmer & Lemeshow test p value shows that neither of the models are good fit but nagelkerke pseudo R² shows that almost 48-49% of variances can be explained by these models. Table 5

**Table 4:** Table showing univariate logistic regression analysis of knowledge, attitude, practice, readiness to manage (n=157).

Variable Name	Category	OR (95% CI)	p-value
**Knowledge Locality**	Rural	Reference	
	Urban	1.9 (1.0, 3.6)	0.04
**Attitude**			
Religion	Other than Hindu	Reference	
	Hindu	2.4 (1.1, 5.5)	0.04
Patients weekly	<60	Reference	
	>60	1.9 (1.1, 3.6)	0.04
Knowledge	Unsatisfactory	Reference	
	Satisfactory	2.1(1.1, 4.0)	0.02
**Practice**			
SES	Below upper	Reference	
	Upper	6.3 (1.0, 38.6)	0.04
**Readiness to manage**			
Locality	Rural	Reference	
	Urban	2.8 (1.4, 5.4)	<0.05
Knowledge	Unsatisfactory	Reference	
	Satisfactory	1.2 (1.1, 2.2)	0.02
Attitude	Unsatisfactory	Reference	
	Satisfactory	1.9 (1.2, 3.8)	0.03

**Table 5:** Table showing multivariable logistic regression analysis of Attitude and readiness to manage domestic violence (n=157).

Variable Name	Category	OR (95% CI)	p-value
**Attitude**			
Religion (Ref cat: Others)	Hindu	1.8 (1.0,3.4)	0.02
Patients weekly (Ref cat: <60)	>60	2.2(1.3,5.2)	0.04
Knowledge (Ref cat: unsatisfactory)	Satisfactory	2.0(1.0,3.9)	<0.05
Nagelkerke R square: (0.49), Hosmer & Lemeshow test: p-value <0.05			
**Readiness to manage**			
Knowledge( Ref cat: unsatisfactory)	Satisfactory	1.5(1.3,2.4)	0.04
Attitude (Ref cat: unsatisfactory)	Satisfactory	2.2(1.0,4.4)	0.02

Nagelkerke R square: (0.48), Hosmer& Lemeshow test: p-value <0.05

## Discussion

Domestic violence remains a prevalent concern in India, significantly affecting the health and overall welfare of women. Within this context, the active involvement of healthcare professionals, particularly Medical intern, is pivotal in recognizing and aiding individuals who are victims of domestic violence. In our study we aimed to assess the knowledge, attitudes, practices, and readiness of intern doctors in a tertiary care medical college and hospital in Kolkata regarding domestic violence.

The study's findings revealed that a substantial number of intern doctors had insufficient knowledge about domestic violence. This is consistent with earlier studies conducted by Renner et al,^29^ Sprague et al. (2013)^30,31^ indicating a recurring issue in understanding this crucial public health concern. This deficiency is a matter of concern, emphasizing the need for improved education and training. The lack of adequate training and education on domestic violence appears to be a significant contributing factor to this knowledge gap. Addressing this deficiency is paramount, and it can be achieved through the integration of domestic violence awareness and intervention components into medical education and training programs. An intriguing finding from the study is that participants from urban areas exhibited a better grasp of domestic violence. This could be attributed to their increased exposure to diversity and a heightened awareness of social issues prevalent in urban settings. The study uncovered that a significant portion of intern doctors held unfavorable attitudes towards domestic violence, a finding consistent with prior research conducted by Renner et al.^29^ The discomfort in inquiring about intimate partner violence (IPV) also resonates with previous studies.^31^ Attitudes are of paramount importance because they can profoundly influence how healthcare providers interact with victims. It's noteworthy that doctors who saw more patients on a weekly basis displayed more positive attitudes, potentially due to their exposure to a wider range of cases and an increased understanding of the significance of addressing domestic violence. This suggests that real-life experiences can have a positive impact on attitudes, a notion supported by findings from Pandey et al.^32^

The study's findings reveal a generally inadequate level of practice among intern doctors when it comes to addressing domestic violence victims. These results align with previous research conducted by Renner et al,^29^ which found that the majority of intern doctors did not engage in any screening practices. Similar patterns were also observed in earlier studies by. Only a small percentage of intern doctors in the current study were able to identify new cases of domestic violence. However, it is worth noting that those who did identify cases primarily offered psychological support, demonstrating a willingness to assist victims. Previous research has shown that healthcare providers who actively screen for intimate partner violence (IPV) and provide counseling can play a vital role in reducing subsequent victimization and improving a patient's overall health. The study revealed that a minority of intern doctors felt adequately prepared to manage domestic violence cases. This deficiency in readiness may stem from the observed overall inadequacy in knowledge and attitudes, a pattern consistent with previous research findings.^29^

Enhancing both knowledge and attitudes emerges as a crucial prerequisite for equipping healthcare professionals with the necessary preparedness to effectively manage domestic violence cases. The study's regression analysis uncovered key factors influencing knowledge, attitudes, and practices concerning domestic violence. Notably, it found that doctors with insufficient knowledge were more likely to exhibit negative attitudes and a lack of readiness to manage such cases. This emphasizes the strong interconnection between knowledge, attitudes, and preparedness. Recognizing this interdependence underscores the importance of addressing both attitudes and knowledge concurrently in medical education and training programs. This finding aligns harmoniously with prior research, which has consistently demonstrated the positive impact of training on improving the identification and referral of survivors of intimate partner violence (IPV) within healthcare settings.^22^

This study emphasizes the urgent need for interventions to enhance Medical interns' knowledge, attitudes, and practices in addressing domestic violence. Participants were often inadequately prepared to identify and respond to domestic violence cases, with their knowledge and preparedness frequently falling below expected standards. To address these deficiencies, medical colleges and institutions must integrate mandatory training modules on domestic violence awareness and intervention into their curricula, aligning with recommendations from previous studies. This approach equips future healthcare professionals with the skills needed to identify and respond to domestic violence cases effectively and empathetically, enhancing both patient outcomes and the healthcare system's overall effectiveness. Additional measures include comprehensive training and education on intimate partner violence within medical, nursing, and dentistry schools, as well as more extensive training for pharmacists and primary care clinicians. Universal Domestic violence screening protocols, especially in rural areas, should be established, emphasizing the recognition of domestic violence indicators, the provision of support to victims, and effective documentation practices to ensure a coordinated response.

Our study's findings highlight the significant implications of preparedness, knowledge, and attitude for adherence to practice guidelines and recommendations. Healthcare professionals should undergo cultural sensitivity training and gain exposure to real-life cases of domestic violence. They should engage in ongoing continuing medical education programs specific to domestic violence, ensuring they are updated on the latest guidelines and best practices. Healthcare systems should establish well-defined protocols and support mechanisms for healthcare professionals to report and address domestic violence cases effectively, including providing psychological support and legal referrals. Regular research and evaluation of healthcare professionals' knowledge, attitudes, and practices related to domestic violence should be conducted to track progress and identify areas requiring improvement, contributing to ongoing enhancement of healthcare responses to domestic violence and better services for victims.

Our study has several limitations to consider. It was conducted at a single tertiary care academic institution, potentially limiting the generalizability of our findings to other healthcare settings. Our focus on Medical Interns provides valuable insights but should be cautiously applied to healthcare providers in different roles. We did not assess the impact of organizational factors or specific residency programs on results, which can be significant contributors to attitudes and preparedness regarding domestic violence. Hypothetical cases in the survey may not fully represent real clinical practice, and self-reported responses may not always reflect actual behaviors. These limitations should be considered when interpreting our study's outcome

Addressing domestic violence in India demands a comprehensive approach, and healthcare providers are integral to this effort. The quality of care for abused women in India has often fallen short due to the various challenges encountered by healthcare professionals. To bridge this significant gap, it is imperative to empower healthcare providers, especially medical interns, with the essential knowledge and training required to sensitively and competently identify and assist victims of domestic violence. This study illuminates the existing shortcomings in the preparedness of intern doctors, offering valuable insights for the development of targeted interventions and enhancements in medical education. These measures aim to equip future healthcare providers with the skills and compassionate attitudes necessary to effectively address this urgent public health issue.

**Acknowledgment**

We are very grateful to Kallol Chatterjee for his valuable input, without whose constant support and inspiration this study would not have been possible. We would like to thank all the experts for taking their valuable time to give feedback of the questionnaire. Lastly we would also like to thank all the participants who despite their busy schedules and academic pressure gave us their valuable and precious time and responded to our mail. 
